# Associations Between High-Sensitivity C-Reactive Protein and All-Cause Mortality Among Oldest-Old in Chinese Longevity Areas: A Community-Based Cohort Study

**DOI:** 10.3389/fpubh.2022.824783

**Published:** 2022-02-08

**Authors:** Pei-Liang Chen, Zhi-Hao Li, Hai-Lian Yang, Zhao-Jin Cao, Xin Cheng, Feng Zhao, Xi-Ru Zhang, Yue-Bin Lv, Fu-Rong Li, Yuan-Feng Zhou, Hao-Nan Li, Ying-Li Qu, Zhao-Xue Yin, Ling Liu, Xian-Bo Wu, Xiao-Ming Shi, Chen Mao

**Affiliations:** ^1^Department of Epidemiology, School of Public Health, Southern Medical University, Guangzhou, China; ^2^Chinese Center for Disease Control and Prevention, National Institute of Environmental Health, Beijing, China

**Keywords:** high-sensitivity C-reactive protein, all-cause mortality, oldest-old, inflammation, aging, cohort study

## Abstract

**Background:**

The association between high-sensitivity C-reactive protein (hsCRP) levels and all-cause mortality for the oldest-old (aged 80 years or older) remains unclear. We aimed to investigate the associations between hsCRP concentrations and the risks of all-cause mortality, and further identify the potential modifying factors affecting these associations among the oldest-old.

**Methods:**

This prospective, community-based cohort study included 2,206 participants aged 80 years or older (median age 93.0 years) from the Healthy Aging and Biomarkers Cohort Study. Cox proportional hazards regression models were used to estimate hazard ratios (HRs) with 95% confidential intervals (95% CIs) for all-cause mortality according to hsCRP quartiles and recommendation for relative risk categories of hsCRP levels (< 1.0, 1.0–3.0, and > 3.0 mg/L), with adjustment for sociodemographic information, lifestyle, physical examination, medical history, and other potential confounders.

**Results:**

During a median follow-up period of 3.1 years (IQR: 1.6–3.9 years), 1,106 deaths were verified. After full adjustment for potential confounders, a higher hsCRP concentration was positively associated with an increased risk of all-cause mortality (*P* for trend < 0.001). Compared with the lowest quartile, the fully adjusted HRs of the second, third, and fourth quartiles were 1.17 (95% CI: 0.94, 1.46), 1.28 (95% CI: 1.01, 1.61), and 1.49 (95% CI: 1.20, 1.87), respectively. The association of hsCRP with all-cause mortality was modified by smoking status (*P* for interaction = 0.011), an increased risk of hsCRP with all-cause mortality showed among non-current smokers (HR: 1.17; 95% CI: 1.07, 1.28), but no significance was observed in current smokers (HR: 0.83; 95% CI: 0.66, 1.18).

**Conclusions:**

Our study indicated that elevated hsCRP concentrations were associated with a higher risk of all-cause mortality among Chinese oldest-old. Future studies investigating additional factors of disease and aging processes are needed to obtain a better understanding of the mechanisms.

## Background

Inflammation has been studied to be the role of a wide range of aging-related diseases (1), such as atherosclerosis and coronary artery disease (2), diabetes (3), Alzheimer's disease (4), and cancer (5). Inflammaging, a description of low-grade, chronic, systemic inflammation in aging, is a highly significant risk factor for both morbidity and mortality in elderly people, as most if not all age-related diseases share inflammatory pathogenesis (6, 7). Nevertheless, the precise etiology of inflammaging and its potential causal role in contributing to adverse health outcomes remain largely unknown (8). Chronic, low-grade elevations in markers of inflammation, such as high-sensitivity C-reactive protein (hsCRP), are potent risk factors for all-cause mortality (9).

CRP, an acute-phase protein produced predominantly by hepatocytes, is a sensitive and exquisitely systemic marker of inflammation (10). CRP has been commonly assayed for infections (11), in-hospital complications (12), prognosis influences (13), and aging-related health outcomes in clinical applications, especially cardiovascular and metabolic disease risk (14, 15). Higher hsCRP levels have been proposed as a predictor of all-cause mortality in many (16–28) but not all studies (29). Inconsistent results may exist due to sex, ethnic or age differences in the populations, and the strength of the association also varied across studies, from 1.14 to 3.64 (hazard ratios or relative risks). Moreover, these findings are based on the general population, but the oldest old adults (octogenarians, non-agenarians, and centenarians) remain underrepresented. The classic risk markers for disease and mortality might not be suitable in the oldest old population (30).

Therefore, we conducted the present study to prospectively examine whether hsCRP was associated with all-cause mortality among the oldest old adults based on datasets from the Healthy Aging and Biomarkers Cohort Study (HABCS).

## Methods

### Design, Study Setting, and Participants

**Figure 1:**
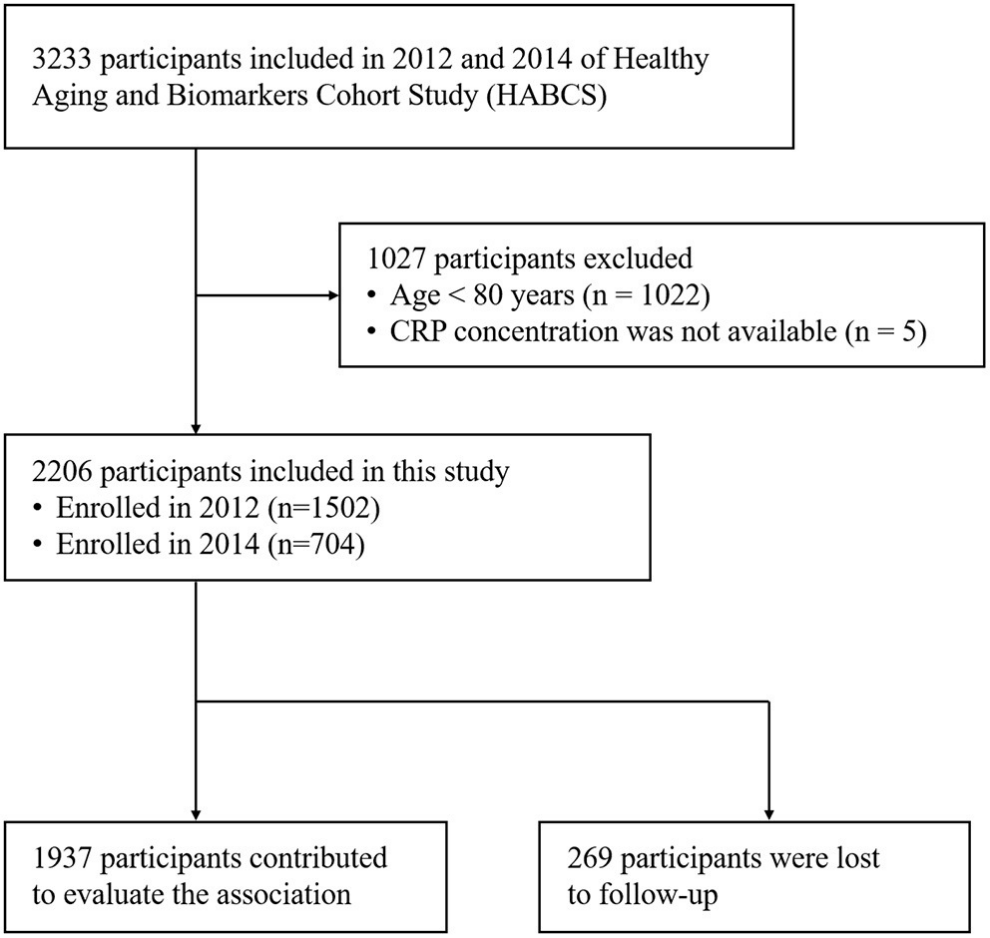
Flowchart of participant enrollment.

This is a prospective, community-based cohort study. Participants were recruited in 2012 and 2014 of HABCS from eight longevity areas selected by the Chinese Society of Gerontology. The densities of oldest old adults are higher (especially for centenarians) in longevity areas than in other areas. These areas include Chen Mai County (Hainan Province), Yong Fu County (Guangxi Province), Ma Yang County (Hunan Province), Zhong Xiang City (Hubei Province), Xia Yi County (He Nan Province), San Shui City (Guangdong Province), Lai Zhou City (Shandong Province), and Ru Dong County (Jiangsu Province). In this study, a total of 2206 participants were enrolled at baseline (1,506 in 2012 and 704 in 2014) and follow-up in 2014 and 2017, respectively. We included all adults aged 80 years or older with available results of CRP tests, and 269 participants were lost to follow-up (Figure 1). The study was approved by the Ethics Committee of Peking University and Duke University. All participants included in HABCS provided informed consent. More details of HABCS have been previously described (31).

### Measurement of hsCRP

Venous blood samples were obtained from the participants by collecting in heparin anticoagulant vacuum tubes, before which participants were required to fast overnight. HsCRP concentration was generally measured through a high-sensitivity immunoturbidimetry assay, and all blood biochemistry tests were conducted by the central clinical lab at Capital Medical University in Beijing. The minimal detectable concentration of hsCRP was 0.11 mg/L.

### Measurement of All-Cause Mortality

We verified the survival status of all participants at baseline during follow-up surveys in 2014 and 2017. The date of death was inquired and ascertained from family members or caregivers of the deceased. The survival time for participants was calculated from the date they enrolled in our study to the date of death. For the survivor, survival time was identified as right-censored at the date of the latest follow-up. Those who could not be found and contacted were recorded as “lost to follow-up.”

### Measurement of Covariates

Covariate information was collected via face-to-face structured questionnaires and biochemistry assays. Covariates in our analyses included sociodemographic information (age, sex, education, and residence), lifestyle (smoking status, alcohol consumption, exercise, and dietary habits), physical examination (body mass index [BMI], systolic blood pressure [SBP], and diastolic blood pressure [DBP]), medical history (hypertension, diabetes, and cardiovascular disease [CVD]), Mini-Mental State Examination (MMSE) score, frailty status, and biochemical indicators (plasma cholesterol, triglycerides, and fasting blood glucose).

Dietary habits include vegetable intake, fruit intake, and meat intake. For the frequencies of food intakes, “almost every day” or “often” were categorized into “often” and “occasionally” and “rarely or never” was categorized into “not often.” For exercise, “yes” or “no” was determined from the question, “Do you do exercises regularly at present?.” MMSE (32) is a practical scale for grading the cognitive state, and the oldest-old in China with MMSE scores below 24 could be defined as having a cognitive impairment (33). Frailty status was classified according to the Study of Osteoporotic Fractures (SOF) index (34) using three components as follows: (1) weight loss (BMI <18.5 kg/m^2^); (2) inability to rise from a chair without using arms; (3) reduced energy level, defined by a “yes” response to the question, “For at least the last 6 months have you been limited in activities people usually do, because of a health problem?”; the status was categorized as robust (no components), prefrail (1 component) or frail (2 or 3 components), which has been shown to be an applicable indicator of biological age in Chinese older adults (35). For the medical history, hypertension was defined as SBP≥140 mmHg and/or DBP≥90 mmHg based on 2018 Chinese guidelines for the management of hypertension (36); diabetes was defined as fasting blood glucose≥7.0 mmol/L based on National guidelines for the prevention and control of diabetes in primary care (37) for the Chinese population; CVD was determined by the self-report of the participants.

### Statistical Analysis

A table for baseline characteristics was generated using descriptive statistics stratified by hsCRP quartiles (mg/L). Continuous data were described by medians and interquartile ranges (IQR), and categorical data were described by frequencies and percentages (%). Hypotheses regarding differences in characteristics across quartiles of hsCRP were analyzed using linear regression for continuous variables and χ^2^-tests for categorical variables.

Kaplan-Meier curves were generated for the quartiles of hsCRP concentrations, and log-rank tests were used to compare different quartile subgroups. Cox proportional hazards regression models were used to estimate hazard ratios (HRs) with 95% confidential intervals (95% CIs) of mortality by hsCRP quartiles, with the lowest quartile (Q1) as the reference group. The Cox models were adjusted for potential confounders that may be associated with both hsCRP concentrations and mortality. The following three models with different adjustments were used: (1) the first model (model 1) tested the association between hsCRP and mortality, controlling for age and sex; (2) the second model (model 2) was further adjusted for other baseline characteristics, namely, education time (0 year or ≥1 year), residence status (rural or urban), smoking status (current or not current), alcohol consumption (current or not current), vegetable intake (often or not often), fruit intake (often or not often), meat intake (often or not often), and exercise (yes or no); and (3) the third adjusted model (model 3) was further adjusted for physical examination, disease status, and biochemical indicators. The aforementioned covariates included BMI (continuous), MMSE scores (continuous), frailty status (frail, prefrail, robust), hypertension (yes or no), diabetes (yes or no), CVD (yes or no), cholesterol (continuous), and triglycerides (continuous). Model 3 was considered to be fully adjusted. Tests of linear trends were performed by treating the median values for each quartile of hsCRP as a continuous variable. A supplementary analysis was also conducted based on the recommendation for relative risk categories of hsCRP levels (38), namely, <1.0 mg/L (low risk), 1.0–3.0 mg/L (average risk), and > 3.0 mg/L (high risk).

The subgroup analyses of HRs for mortality by each 10 mg/L increase in hsCRP were performed according to sex (men or women), age (80+ years, 90+ years, and 100+ years), education, residence, smoking status, alcohol consumption, fruit intake, meat intake, vegetable intake, exercise, BMI (<18.5 kg/m^2^, ≥18.5 and <24 kg/m^2^, ≥24 kg/m^2^), MMSE (<24 or ≥24), frailty status. Possible interaction effects were explored by groups from the abovementioned characteristics and a likelihood ratio test was performed to test for interactions by comparing the statistical fit of models with and without interaction terms in the fully adjusted model.

We also conducted sensitivity analyses to examine the robustness of our findings: (1) to exclude participants who died during the first 1 year of follow-up; (2) participants were divided by tertiles and quintiles of hsCRP concentrations.

Analyses were conducted using Stata version 14.0 (College Station, Texas). A *P* < 0.05 was considered statistically significant.

## Results

### Baseline Characteristics

Among 2,206 individuals, the median age of participants was 93 years (IQR: 86-100 years). A total of 1,417 were women (64.23%), and 1,905 were living in rural areas (87.19%). Baseline characteristics are summarized in Table 1 by hsCRP quartiles. The median hsCRP concentration was 1.13 mg/L (IQR: 0.46–2.92 mg/L), with no significant associations with age or residence. Of those in the highest quartile of hsCRP, a greater proportion of the adults were women and frail, inclined to have less fruit and meat intakes, reported to have no hypertension or CVD, and tended to have lower levels of cholesterol and glucose.

**Table 1:** Characteristics of participants by quartiles of high-sensitivity C-reactive Protein.

	**Overall**	**High-sensitivity C-reactive Protein^a^ (mg/L)**				***P***
		**Q1**	**Q2**	**Q3**	**Q4**	
		**≥0.46**	**0.47–1.13**	**1.14–2.92**	**≥2.93**	
No. of participants	2,206	564	547	544	551	
Age, median (IQR), years	93 (86, 100)	93 (87, 100)	92 (85, 100)	92 (86, 100)	94 (88, 100)	0.389
Women, *n* (%)	1,417 (64.23)	405 (71.81)	344 (62.89)	336 (61.76)	332 (60.25)	<0.001
Residence, *n* (%)						0.102
Urban	280 (12.81)	64 (11.43)	58 (10.70)	78 (14.42)	80 (14.76)	
Rural	1,905 (87.19)	496 (88.57)	484 (89.30)	463 (85.58)	462 (85.24)	
Education time, years						0.706
0	1,714 (78.41)	444 (79.86)	417 (76.94)	424 (78.23)	429 (78.57)	
≥1	472 (21.59)	112 (20.14)	125 (23.06)	118 (21.77)	117 (21.43)	
Smoking status, *n* (%)						0.986
Current	220 (10.22)	56 (10.13)	53 (9.98)	54 (10.13)	57 (10.63)	
Not current	1,933 (89.78)	497 (89.87)	478 (90.02)	479 (89.87)	479 (89.37)	
Alcohol drinking status, *n* (%)						0.780
Current	254 (11.81)	68 (12.34)	67 (12.64)	61 (11.47)	58 (10.80)	
Not current	1,896 (88.19)	483 (87.66)	463 (87.36)	471 (88.53)	479 (89.20)	
Frequent vegetable intake, *n* (%)^b^	1,240 (57.57)	309 (55.98)	325 (61.21)	315 (59.10)	291 (54.09)	0.085
Frequent fruit intake, *n* (%)^b^	795 (36.75)	223 (40.11)	205 (38.46)	203 (37.94)	164 (30.43)	0.005
Frequent meat intake, *n* (%)^b^	1,024 (48.51)	251 (45.72)	271 (52.62)	277 (53.47)	225 (42.53)	<0.001
Habitual exercise, *n* (%)^c^	263 (12.51)	82 (15.27)	63 (12.14)	61 (11.66)	57 (10.90)	0.146
Medical history						
Hypertension, *n* (%)	841 (38.12)	254 (45.04)	201 (36.75)	191 (35.11)	195 (35.39)	<0.001
Diabetes, *n* (%)	189 (8.57)	34 (6.03)	38 (6.95)	58 (10.66)	36 (6.53)	0.005
CVD, *n* (%)	311 (14.10)	71 (12.59)	69 (12.61)	83 (15.26)	88 (15.97)	0.405
Frailty						0.002
Frail, *n* (%)	740 (33.54)	194 (34.40)	159 (29.07)	168 (30.88)	219 (39.75)	
Prefrail, *n* (%)	753 (34.13)	193 (34.22)	189 (34.55)	186 (34.19)	185 (33.58)	
Robust, *n* (%)	713 (32.32)	177 (31.38)	199 (36.38)	190 (34.93)	147 (26.68)	
MMSE scores, median (IQR)	25 (18, 28)	25 (17, 28)	26 (19, 28)	26 (19, 28)	24 (16, 28)	0.321
BMI, median (IQR), kg/m^2^	20.00 (17.78, 22.81)	19.48 (17.58, 22.03)	20.41 (18.29, 23.45)	20.34 (18.03, 22.96)	19.93 (17.78, 22.75)	0.461
Systolic pressure, median (IQR), mmHg	140 (126, 160)	143 (130, 160)	140 (128, 160)	141.5 (127.5, 160)	140 (121, 155.5)	<0.001
Total cholesterol, median (IQR), mmol/L	4.31 (3.67, 5.02)	4.38 (3.68, 5.02)	4.33 (3.75, 5.02)	4.43 (3.78, 5.17)	4.07 (3.5, 4.83)	0.014
Triglycerides, median (IQR), mmol/L	0.86 (0.64, 1.19)	0.83 (0.61, 1.12)	0.88 (0.66, 1.23)	0.92 (0.67, 1.28)	0.84 (0.63, 1.17)	0.132
Glucose, median (IQR), mmol/L	4.68 (4.00, 5.45)	4.48 (3.89, 5.17)	4.71 (4.07, 5.45)	4.80 (4.10, 5.54)	4.69 (3.96, 5.67)	0.001

*hsCRP, high-sensitivity C-reactive protein; CVD, cardiovascular disease; MMSE, Mini-Mental State Examination; BMI, body mass index*.

a *Quartiles of hsCRP: median (IQR), mg/L*.

b *“Frequent intake” was defined by the frequencies of “almost every day” or “often”*.

a *“Habitual exercise” was defined as “exercise at present”*.

### HsCRP and All-Cause Mortality

**Figure 2:**
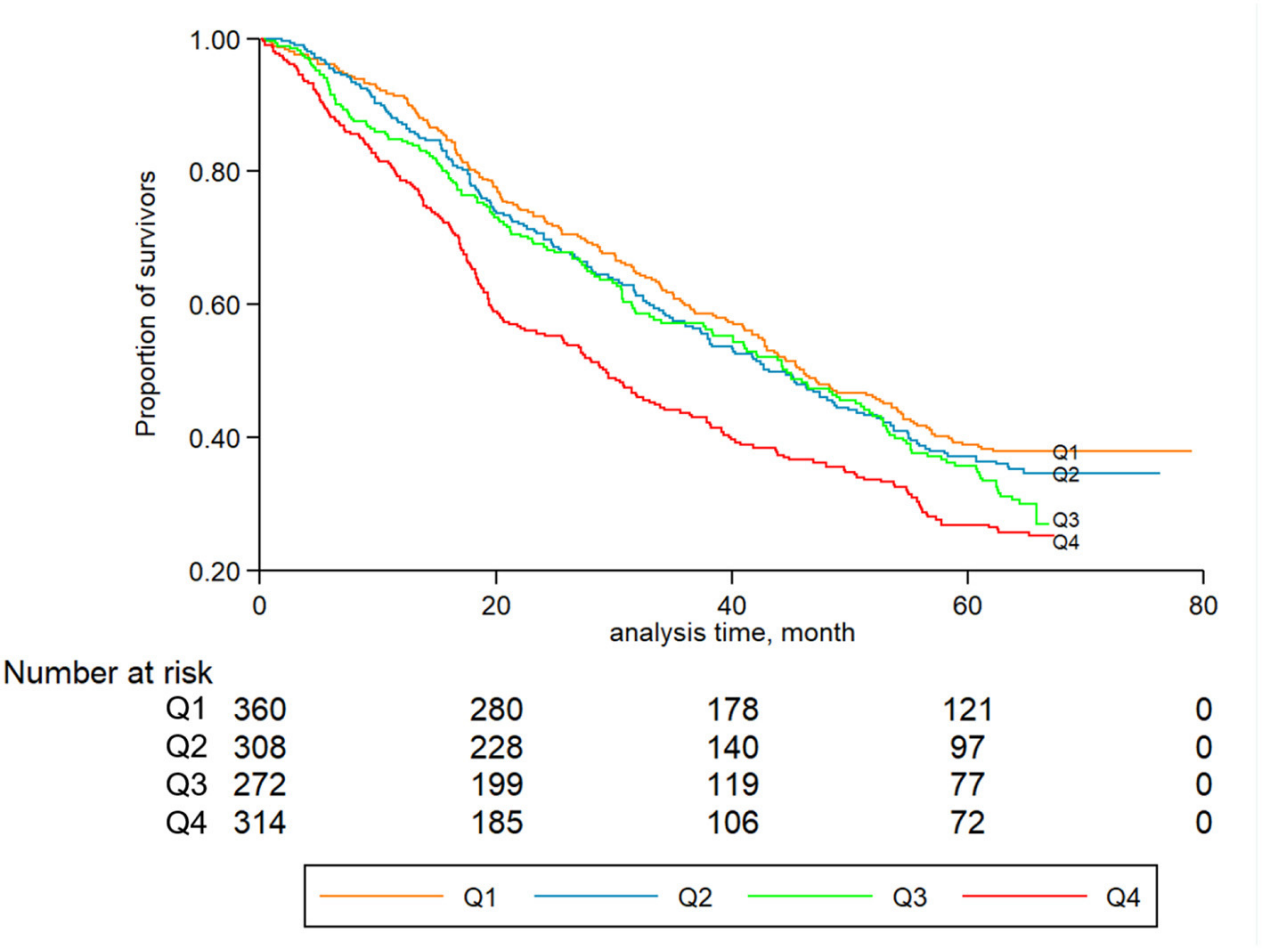
Kaplan-Meier graphs for all-cause mortality by quartiles of CRP.

During a median follow-up period of 3.1 years (IQR: 1.6–3.9 years), a total of 1,106 all-cause deaths occurred (men: 380; women: 726). Figure 2 displays the Kaplan-Meier curves for all-cause mortality by quartiles of hsCRP. The log-rank tests showed significant differences in all-cause mortality among different levels of hsCRP (*P* < 0.001). Table 2 presents the association between hsCRP and mortality. Compared to the lowest quartile, the fully adjusted HRs of the second, third, and fourth quartiles were 1.17 (95% CI: 0.94, 1.46), 1.28 (95% CI: 1.01, 1.61), and 1.49 (95% CI: 1.20, 1.87), respectively (Table 2). The risk of all-cause mortality increased with elevated hsCRP (*P* < 0.001). Compared to those with hsCRP <1.0 mg/L, individuals with hsCRP > 3.0 mg/L had a significantly higher risk (HR: 1.39; 95% CI: 1.14, 1.70) of all-cause mortality even after full adjustment.

**Table 2:** Association between hsCRP and all-cause mortality.

	**Deaths/** ***N***	**HR [95% CI]^a^ for all-cause mortality**		
		**Model 1**	**Model 2**	**Model 3**
**Quartiles of hsCRP**				
Q1	263/491	1.00 (reference)	1.00 (reference)	1.00 (reference)
Q2	264/493	1.15 (0.94, 1.40)	1.10 (0.89, 1.36)	1.17 (0.94, 1.46)
Q3	271/483	1.27 (1.03, 1.55)	1.24 (1.00, 1.53)	1.28 (1.01, 1.61)
Q4	308/470	1.53 (1.27, 1.86)	1.50 (1.23, 1.84)	1.49 (1.20, 1.87)
*P*-trend		<0.001	<0.001	<0.001
**Levels of hsCRP**				
<1.0 mg/L	491/911	1.00 (reference)	1.00 (reference)	1.00 (reference)
1–3.0 mg/L	312/565	1.17 (0.98, 1.39)	1.18 (0.98, 1.41)	1.17 (0.96, 1.42)
>3.0 mg/L	303/461	1.45 (1.22, 1.71)	1.44 (1.21, 1.73)	1.39 (1.14, 1.70)
*P*-trend		<0.001	<0.001	0.001

a *HR, hazard ratio; CI, confidence interval*.

*Model 1: adjusted for age, sex*.

*Model 2: adjusted for age, sex, education, residence, smoking, drinking, exercise, fruit intake, meat intake, vegetable intake*.

*Model 3: adjusted for age, sex, education, residence, smoking, drinking, exercise, fruit intake, meat intake, vegetable intake, BMI, MMSE, frailty, hypertension, diabetes, CVD, cholesterol, triglycerides*.

### Subgroup Analyses

Subgroup analyses stratified by major confounders are presented in Table 3. The HRs showed similar results with no significant differences across most subgroups defined by age, sex, education, residence, drinking status, vegetable intake, fruit intake, meat intake, exercise, BMI, frailty status, MMSE scores (all P for interaction > 0.05). However, a significant interaction from smoking status was noted (*P* = 0.011), where an increased risk of hsCRP with all-cause mortality showed among non-current smokers (HR: 1.17; 95% CI: 1.07, 1.28), but no significance was observed in current smokers (HR: 0.83; 95% CI: 0.66, 1.18).

**Table 3:** Subgroup analyses for the hazard ratio of all-cause mortality for each 10 mg/L increase in hsCRP.

**Subgroup**	**HR [95%CI]^a^**	**P for interaction**
Age		0.214
80+ years	1.02 (0.85, 1.23)	
90+ years	1.04 (0.93, 1.16)	
100+ years	1.23 (1.05, 1.45)	
Sex		0.135
Women	1.16 (1.04, 1.29)	
Men	0.98 (0.87, 1.10)	
Education time		0.226
0 year	1.11 (1.02, 1.20)	
≥1 year	0.92 (0.68, 1.24)	
Residence		0.418
Urban	0.95 (0.76, 1.18)	
Rural	1.10 (1.01, 1.19)	
Smoking status		0.011
Current	0.83 (0.66, 1.03)	
Not current	1.17 (1.07, 1.28)	
Drinking status		0.446
Current	0.98 (0.68, 1.40)	
Not current	1.08 (0.10, 1.17)	
Habitual exercise		0.820
Yes	1.02 (0.63 1.67)	
No	1.08 (1.00, 1.17)	
Vegetable intake		0.289
Often	1.16 (1.00, 1.34)	
Not often	1.05 (0.95, 1.16)	
Fruit intake		0.125
Often	1.18 (0.98, 1.43)	
Not often	1.03 (0.94, 1.13)	
Meat intake		0.646
Often	1.07 (0.89, 1.29)	
Not often	1.09 (1.00, 1.18)	
BMI		0.798
<18.5	1.14 (0.98, 1.32)	
≥18.5 and <24	1.04 (0.94, 1.16)	
≥24	0.99 (0.73, 1.35)	
MMSE scores		0.556
<24	1.10 (1.00, 1.21)	
≥24	1.05 (0.91, 1.21)	
Frailty		0.784
Frail	1.08 (0.95, 1.22)	
Prefrail	1.10 (0.97, 1.25)	
Robust	1.00 (0.81, 1.23)	

a *HR, hazard ratio; CI, confidence interval*.

*HRs were adjusted for age, sex, education, residence, smoking, drinking, exercise, fruit intake, meat intake, vegetable intake, BMI, MMSE, frailty, hypertension, diabetes, CVD, cholesterol, triglycerides*.

### Sensitivity Analyses

In the sensitivity analyses, we did not find notable changes in the results after excluding deaths during the first 1 year of follow-up (Supplementary Table 1). Similar associations and trends were observed when participants were divided by tertiles (Supplementary Table 2) and quintiles (Supplementary Table 3) of hsCRP concentrations.

## Discussion

In this population-based study of the oldest old adults living in Chinese longevity areas, the participants with higher hsCRP concentrations had an increased risk of mortality, even after adjusting for potential confounders. The association of hsCRP with all-cause mortality was less likely to be modified by sociodemographic factors, physical examinations, biochemical indicators and most lifestyle factors, except for smoking status.

Our findings are consistent with previous studies demonstrating positive associations between hsCRP and all-cause mortality, which are significant at higher levels of the hsCRP distribution (16, 20–22, 24–26, 28). The estimated value might differ by ethnicity because Asian populations tend to have a lower hsCRP level than Western populations (39, 40). A study (41) with 11,623 middle-aged Chinese individuals categorized three groups based on hsCRP levels (< 1.0, 1.0–3.0, and > 3.0 mg/L) and obtained the result that the HR for all-cause mortality in the > 3.0 group was 2.64 (95% CI: 1.74, 4.01). This difference inferred that the estimate might wane with age or that the sample size of our current study was not sufficient to provide power to detect a difference.

It is also important to note that sex was not a modifier in our study. Some studies showed a positive association in both sexes (9, 24, 27), while significant differences appeared to exist in a single sex [mostly men (29, 42, 43)]. However, whether men or women were at a greater risk remains controversial (9, 24, 43, 44). The differential effect of hsCRP in predicting all-cause mortality risk by sex warrants further investigation. Smoking status is another novel point in which significant interaction was found. However, the estimate seemed to be stronger in non-smokers, though both stratifications showed no significant differences. One possibility is that inflammation adaption might occur in the human body during the period of habitual smoking, resulting in a lower hazard to current smokers than to those who did not smoke during the same period. Evidence showed that smoking cessation does not reduce CRP (45), or the time from smoking cessation may influence the concentrations (46). From another consideration, a limited sample size leads to a lack of statistical significance in the estimate, and even interaction exists. But in our study, we did not take this into account during grouping, which might cause misclassification bias. The interaction observed by smoking status warrants further research.

Potential limitations of the current study should be considered in evaluating our results. Our study was observational in nature, and we cannot rule out the possibility of reverse causality; therefore, hsCRP might also be a consequence of diseases rather than a cause. Moreover, the residual confounded by other unmeasured or unknown factors likely existed and potentially results were biased in an unknown direction despite our full adjustment in analyses, such as chronic diseases, which were self-reported thus residual confounding may still exist. The association might be partly affected by the loss to follow-up, which required further studies to verify this association. Additionally, since information on the subtype of death was not collected in the HABCS, in-depth analyses based on cause-specific mortality are necessary but unable to conduct. Finally, similar to most other studies, the fact that hsCRP was measured only once at baseline is a potential limitation because random fluctuation in this parameter over time would tend to increase the variance in the data; how trajectories of hsCRP may influence mortality remains undetermined.

Despite these limitations, this study has noteworthy strengths when compared to prior research. Above all, our findings were based on a prospective study with integrated and detailed baseline, outcome, and blood sample data. The robustness of the outcomes measured and the large sample size of the oldest old adults increases the relevance of our findings. For representativeness, it is believed that community-dwelling older adults are more typical due to the dominance of family care in Chinese society. A distinguishing feature of this study is that all of the longevity areas we investigated provided a distinct population of oldest old adults, which broadens the evidence from existing research with a unique age spectrum.

## Conclusion

Our analyses indicated that elevated hsCRP concentrations are associated with a higher risk of all-cause mortality among the oldest-old adults. Future studies investigating additional factors of disease and aging processes are needed to conduct a better understanding of the mechanisms.

## Data Availability Statement

The raw data supporting the conclusions of this article will be made available by the authors, without undue reservation.

## Ethics Statement

Written informed consent was obtained from the individual(s) for the publication of any potentially identifiable images or data included in this article.

## Author Contributions

CM, X-MS, and X-BW designed the study analysis. Z-JC, FZ, Y-BL, Y-LQ, and LL conducted HABCS and directed its implementation, including quality assurance and control, dataset management, and analytic strategy. Z-HL and F-RL contributed to data cleaning. Y-BL and Z-XY helped supervise the field activities. P-LC and Z-HL designed the study's analytic strategy, performed the statistical analyses, and had primary responsibility for writing the manuscript. P-LC, Z-HL, H-LY, XC, Y-FZ, and H-NL analyzed the data and prepared the manuscript. CM and X-MS are guarantors of the paper. All authors have critically commented on, revised the manuscript, and approved the final version.

## Funding

This work was supported by the National Natural Science Foundation of China (81973109), the Project Supported by Guangdong Province Universities and Colleges Pearl River Scholar Funded Scheme (2019), the National Key Research and Development Program of China (2018YFC2000400), the Special Fund for Scientific and Technological Innovation Strategy of Guangdong Province (Climbing Program) (pdjh2021b0105) and the Innovation and Entrepreneurship Training Program for College Students (S202012121105).

## Conflict of Interest

The authors declare that the research was conducted in the absence of any commercial or financial relationships that could be construed as a potential conflict of interest.

## Publisher's Note

All claims expressed in this article are solely those of the authors and do not necessarily represent those of their affiliated organizations, or those of the publisher, the editors and the reviewers. Any product that may be evaluated in this article, or claim that may be made by its manufacturer, is not guaranteed or endorsed by the publisher.

## Abbreviations

BMI: body mass index
CI: confidential interval
HABCS: Healthy Aging and Biomarkers Cohort Study
hsCRP: high-sensitivity C-reactive protein
CVD: cardiovascular disease
DBP: diastolic blood pressure
HR: hazard ratio
IQR: interquartile range
MMSE: Mini-Mental State Examination
SBP: systolic blood pressure
SOF: Study of Osteoporotic Fractures.
