# Supplementary material for: Associations Between High-Sensitivity C-Reactive Protein and All-Cause Mortality Among Oldest-Old in Chinese Longevity Areas: A Community-Based Cohort Study
Source: Front Public Health. 2022 Feb 8;10:824783. doi: 10.3389/fpubh.2022.824783 (PMC8861080; doi:10.3389/fpubh.2022.824783)
Supplement: Supplementary file 1 [file Table_1.docx]

**Supplementary Table 1** Association between hsCRP and all-cause mortality after excluding deaths during the first 1 year of follow-up

|  | deaths/N | HR [95% CI]^a^ for all-cause mortality | | |
| --- | --- | --- | --- | --- |
|  |  | Model 1 | Model 2 | Model 3 |
| Risk by quartiles after excluding deaths during the first 1 year of follow-up |  |  |  |  |
| Q1 | 207/430 | 1.00 (reference) | 1.00 (reference) | 1.00 (reference) |
| Q2 | 202/417 | 1.13 (0.91, 1.42) | 1.14 (0.91, 1.45) | 1.18 (0.92, 1.52) |
| Q3 | 204/425 | 1.19 (0.95, 1.50) | 1.18 (0.93, 1.50) | 1.23 (0.96, 1.59) |
| Q4 | 226/398 | 1.33 (1.07, 1.65) | 1.30 (1.03, 1.63) | 1.35 (1.05, 1.73) |
| P-trend |  | 0.010 | 0.028 | 0.018 |
| Levels of hsCRP |  |  |  |  |
| <1.0 mg/L | 397/817 | 1.00 (reference) | 1.00 (reference) | 1.00 (reference) |
| 1-3.0 mg/L | 232/485 | 1.06 (0.87, 1.29) | 1.05 (0.85, 1.29) | 1.08 (0.87, 1.34) |
| >3.0 mg/L | 210/368 | 1.28 (1.05. 1.55) | 1.26 (1.03, 1.56) | 1.29 (1.04, 1.62) |
| P-trend |  | 0.019 | 0.035 | 0.027 |

^a^ HR: hazard ratio; CI: confidence interval.

HRs were adjusted for age, sex, education, residence, smoking, drinking, exercise, fruit intake, meat intake, vegetable intake, BMI, MMSE, frailty, hypertension, diabetes, CVD, cholesterol, triglycerides.

**Supplementary Table 2** Risk of all-cause mortality by tertiles of hsCRP concentrations

|  | deaths/N | HR [95% CI]^a^ for all-cause mortality | | |
| --- | --- | --- | --- | --- |
|  |  | Model 1 | Model 2 | Model 3 |
| Tertiles of hsCRP |  |  |  |  |
| T1 | 359/651 | 1.00 (reference) | 1.00 (reference) | 1.00 (reference) |
| T2 | 342/651 | 1.16 (0.97, 1.39) | 1.15 (0.96, 1.39) | 1.13 (0.93, 1.38) |
| T3 | 405/635 | 1.42 (1.20, 1.68) | 1.39 (1.17, 1.66) | 1.34 (1.04, 1.63) |
| P-trend |  | <0.001 | <0.001 | 0.003 |

^a^ HR: hazard ratio; CI: confidence interval.

HRs were adjusted for age, sex, education, residence, smoking, drinking, exercise, fruit intake, meat intake, vegetable intake, BMI, MMSE, frailty, hypertension, diabetes, CVD, cholesterol, triglycerides.

**Supplementary Table 3** Risk of all-cause mortality by quintiles of hsCRP concentrations

|  | deaths/N | HR [95% CI]^a^ for all-cause mortality | | |
| --- | --- | --- | --- | --- |
|  |  | Model 1 | Model 2 | Model 3 |
| Quintiles of hsCRP |  |  |  |  |
| Q1 | 209/396 | 1.00 (reference) | 1.00 (reference) | 1.00 (reference) |
| Q2 | 219/388 | 1.17 (0.94, 1.46) | 1.15 (0.92, 1.46) | 1.27 (0.99, 1.62) |
| Q3 | 204/386 | 1.35 (1.07, 1.70) | 1.30 (1.02, 1.67) | 1.36 (1.04, 1.77) |
| Q4 | 224/396 | 1.17 (0.94, 1.47) | 1.17 (0.92, 1.49) | 1.32 (1.01, 1.71) |
| Q5 | 250/371 | 1.69 (1.38, 2.09) | 1.65 (1.32, 2.07) | 1.64 (1.28, 2.10) |
| P-trend |  | <0.001 | <0.001 | <0.001 |

^a^ HR: hazard ratio; CI: confidence interval.

HRs were adjusted for age, sex, education, residence, smoking, drinking, exercise, fruit intake, meat intake, vegetable intake, BMI, MMSE, frailty, hypertension, diabetes, CVD, cholesterol, triglycerides.
